# The vascularized periosteum flap as novel tissue engineering model for repair of cartilage defects

**DOI:** 10.1111/jcmm.12485

**Published:** 2015-03-05

**Authors:** Leila Harhaus, Jung-Ju Huang, Shu-Wei Kao, Yen-Lin Wu, Gina Alicia Mackert, Bernd Höner, Ming-Huei Cheng, Ulrich Kneser, Chao-Min Cheng

**Affiliations:** a Department of Hand-, Plastic and Reconstructive Surgery, Burn Care Center, Department of Plastic Surgery of Heidelberg University, BG Traumacenter LudwigshafenLudwigshafen, Germany; b Department of Plastic and Reconstructive Surgery, Chang Gung Memorial Hospital, Chang Gung University, College of MedicineTaoyuan, Taiwan; c Institute of Nanoengineering and Microsystems, National Tsing Hua UniversityHsinchu, Taiwan; d Department of Social and Legal Sciences, SRH UniversityHeidelberg, Germany

**Keywords:** cartilage repair, tissue engineering, vascularized periosteum flap, translational research, osteoarthritis, cartilage defects

## Abstract

Periosteum is a promising tissue engineering scaffold in research of cartilage repair; so far however, periosteum transfers have not been realized successfully because of insufficient nourishment of the graft. In a translational approach we, for the first time, designed a vascularized periosteum flap as ‘independent’ biomaterial with its own blood supply to address this problem and to reconstruct circumscript cartilage defects. In six 3-month-old New Zealand rabbits, a critical size cartilage defect of the medial femur condyle was created and covered by a vascularized periosteum flap pedicled on the saphenous vessels. After 28 days, formation of newly built cartilage was assessed macroscopically, histologically and qualitatively via biomechanical compression testing, as well as on molecular biological level via immunohistochemistry. All wounds healed completely, all joints were stable and had full range of motion. All flaps survived and were perfused through their pulsating pedicles. They showed a stable attachment to the bone, although partially incomplete adherence. Hyaline cartilage with typical columnar cell distribution and positive Collagen II staining was formed in the transferred flaps. Biomechanical testing revealed a significantly higher maximum load than the positive control, but a low elasticity. This study proved that vascularization of the periosteum flap is the essential step for flap survival and enables the flap to transform into cartilage. Reconstruction of circumscript cartilage defects seems to be possible. Although these are the first results out of a pilot project, this technique, we believe, can have a wide range of potential applications and high relevance in the clinical field.

## Introduction

As articular cartilage possesses neither a blood supply nor a source of mesenchymal stem cells (MSCs), it has a limited potential to repair itself when damaged or diseased 1. MSCs in periosteum have been shown to differentiate into neochondrocytes *in vivo* and *in vitro* 2–5 with the potential to form cartilage in the presence of specific preconditions, such as transforming growth factor-beta 1 (TGF-β1) or shear stress 6,7. This is the scientific basis for autologous periosteal grafting (periosteal arthroplasty) as a treatment option to repair defects in articular cartilage.

To date, osteoarthritis of the knee is still a not curable disease. Standard surgical techniques contain the arthroscopic debridement, autologous transplantation of chondrocytes, transposition osteotomies and partial or total joint replacements. Periosteum is a promising tissue in cartilage regeneration research because of several aspects. One advantage is that periosteal tissue meets the three requirements of tissue engineering. First, because periosteal tissue can be transplanted as a whole tissue, it can serve as its own scaffold or matrix. Second, it presents a source of pluripotent MSCs with the potential to form cartilage 8 and third it produces bioactive factors that enhance cell growth and differentiation. Numerous growth factors involved in regulating chondrocytes and cartilage development are synthesized by periosteum in conditions conductive to chondrogenesis. These include TGF-β1, insulin-like growth factor-1, growth and differentiation factor-5, bone morphogenetic protein-2, integrins, and the receptors for these molecules 8.

Regarding various previously utilized models within this promising field of cartilage engineering through periosteal tissue, there are several *in vivo* models with different designs of tissue transfer. All of them, however, work with free, non-vascularized periosteum transfer, resulting in incomplete filling of the defect and the development of mostly fibrous tissue instead of hyaline cartilage 9. As adequate nutrition of transferred tissue is an indispensable precondition and in case of periosteal tissue may not only occur through synovial fluid, we consider a vascularized periosteal flap model to be the next necessary step in joint cartilage repair.

With this project, we want to combine the knowledge and experience gained through plastic surgery concerning regeneration and reconstruction of injured tissue with the common orthopaedic disease of degenerative cartilage defects and arthritis.

We want to proof our hypothesis that tissue nourishment through a dedicated pedicle is the most important aspect for survival and transformation of the transferred periosteal tissue. With the new technique of using a vascularized periosteum flap, we want to establish a new approach and surgical model for tissue-engineered cartilage regeneration and go the first step towards the creation of a physiological, healthy and weight-bearing new cartilage developed from autologous material.

## Material and methods

The study design was approved by the ethical committee of Chang Gung Memorial Hospital and all animal procedures complied with the Chang Gung Memorial Hospital animal research guidelines.

### Preparatory work

Before starting the presented *in vivo* study, we developed and validated the surgical concept precisely. On six rabbit cadavers, we carefully performed and evaluated the necessary surgical steps, including the harvesting technique of the periosteum flap, the creation of a purely cartilage defect without touching the subchondral bone, the preparation of the pedicle and rotation of the flap into the knee and in particular, the fixation technique of the flap onto the defect.

### Animals

For the *in vivo* study, six 3-month-old New Zealand rabbits (Livestock Research Institute, Tainan, Taiwan), weighing approximately 2.5 kg were used under the guidelines of Animal Research Committee of Chang Gung Memorial Hospital. The rabbits were kept at temperature of 17–23°C with 30–80% humidity and light-dark 12:12 hr cycles with free access to water and standard chow.

### Surgery and groups

Surgeries were performed in narcosis using Zoletil© with Rompun© (Xylazine Hydrochloride 23.32 mg/ml) in a ratio of 1:1 and injections of 2.3 ml for a 3.0 kg rabbit. The Zoletil^©^ was supplied in a sterile vial as a lyophilized powder containing 125 mg of tiletamine and 125 mg of zolazepam and 5 ml of distilled water.

**Figure 1:**
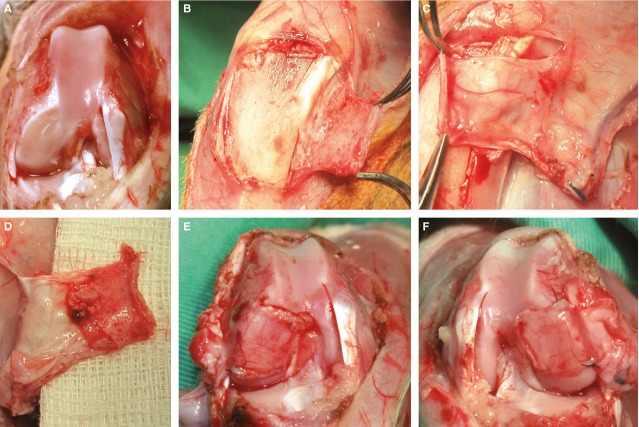
Surgical technique. (A) Left leg after creation of a critical size cartilage defect of the medial and lateral condyle. (B) Cautious separation of the periosteum flap from the bony surface. (C) Harvesting of the periosteum flap based on the saphenous artery and its venae comitantes, the nourishing vessel to the periosteum can be seen clearly. (D) Bottom surface of the raised flaps; the cambium layer is visible. (E and F) Right and left leg after coverage of the medial defects and fixation with transosseous sutures.

After shaving of the hindlimb, it was scrubbed to sterility with polyvidone iodine and the extremity was then covered with a sterile sheet. Then, a longitudinal incision along the medial parapatellar line and ventral tibia was performed. After preparation of subcutaneous tissue under cautious haemostasis and parapatellar incision of the medial capsule the patella was dislocated laterally to expose the knee joint. A full-thickness cartilage defect of 4 × 4 mm [critical size defect International Cartilage Repair Society (ICRS) grade IV] was created in the lateral and the medial femur condyle in both legs using a rotating grinding disc. Care was taken to avoid subchondral bone injury, which was confirmed by complete absence of bleeding, to prevent a possible local cartilage recovery from ingrowing bone marrow stem cells. With this technique, four defects per animal could be achieved. The defects of the medial condyles of both sides were covered with the periosteum flaps, the defects of the lateral condyles of both sides were not treated and served as negative control (Fig.1A). (After sacrifice, the dorsal aspects of the lateral condyles were additionally harvested to serve as positive controls.)

An axial pattern tibia periosteal flap based on the saphenous artery and its venae comitantes (Fig.1B–D) was harvested along with a perivascular tissue cuff (average size, 5 mm × 15 mm). The pedicle was dissected according to the surgical technique of Chen *et al*., who performed this flap design for osteogenesis examination 10. While harvesting of the periosteal flaps, special care was taken on the exact preparation and separation of the cambium layer from the bony surface, because chondrocyte precursor cells located in this layer have to be completely included in the flap. This technique has the advantage of free arc of rotation and therefore free positioning of the flap into the defect (Fig.1E and F). The flap was then fixed into the lesion site using 6.0 ethilon suture in a transosseous suture technique. The fixation was furthermore secured with application of Tissucol Duo S^©^ two components fibrinogen tissue glue, which we figured out to be useful during our preparatory work. After flap fixation, the patella was repositioned and the incised joint capsule was closed with a 4-0 absorbable suture (PDS) except for a small triangular space containing the flap pedicle. With passive movement of the joint it was ensured, that there was no compression on the pedicle in any joint position. The superficial fascia and skin was sutured with 4-0 nylon materials.

For post-operative analgesia, rabbits received Ketoprofen 5 mg/kg, daily i.m. injections, for 5 days post-operatively. After surgery, rabbits were allowed to move freely in their cages and exercise their legs with full weight bearing of the knee.

The healing period was assessed for 4 weeks.

### Euthanasia and specimen collection

Four weeks post-operatively, a video-assisted macroscopical gait analysis was performed. Animals were allowed to move free in a 3 × 3 m field with a rough textile floor for 30 min. After a habituation period of 10 min., use of the legs (complete/incomplete), weight bearing (full/restrictive posture) and walking patterns (harmonic/hobbling) were assessed by a commercial video-setting macroscopically.

After that, the rabbits were narcotized again for specimen collection (it was important to evaluate the joint in the live animal to assess the pulsation of the pedicle). Wound appearance, range of motion of the joints and joint stability in all directions was performed controlling the collateral ligaments and cruciate ligaments.

For preparation, the surgical site was opened again and checked for signs of infection or fluid collection. The periost-harvesting defect of the ventral tibia diaphysis was examined macroscopically in terms of regeneration of the periosteum or possible partial necrosis of the cortical tibia bone because of reduced blood supply.

Concerning the joint, first the pulsation of the saphenous pedicle was checked as sign of the viability of the flap. Then, the joint was opened in the same way again and the appearance of the condyle's surfaces was photographed and evaluated in terms of flap attachment and macroscopic surface quality. Visually acceptable repairs were noted as smooth, firm repair tissue that filled the defects.

The medial and lateral, as well as the dorsolateral femur condyles of both knees were then harvested along the frontal plane and fixed for histological examination in 4% buffered formalin. The rabbits were then killed by an overdose injection of lidocaine.

### Histology

A knee of each rabbit was randomly selected to undergo histological and immunohistochemical analyses – they were decalcified in 10% nitric acid for at least 2 weeks and then dehydrated and embedded in paraffin according to standard methods, then they were sectioned and processed for routine haematoxylin and eosin and Movat's staining, also according to standard methods for evaluation of cartilage specific extra cellular matrix. The quality of regenerated tissue in the articular cartilage defect in different groups was scored according to the ICRS scale (Table1) 11. Each section was examined and scored separately. Sections were graded according to: (*i*) surface continuity; (*ii*) matrix staining; (*iii*) integration of regenerative tissue with surrounding articular cartilage; (*iv*) chondrocyte morphology; (*v*) cartilage thickness and (*vi*) subchondral bone structure.

**Table 1:** Modified ICRS histological scoring scale for evaluation of articular cartilage repair [11]

Feature	Score
Surface	
Smooth/continuous	3
Discontinuous/irregularities	0
Matrix	
Hyaline	3
Mixture: hyaline/fibrocartilage	2
Fibrocartilage	1
Fibrous tissue	0
Cell distribution	
Mixed/columnar clusters	3
Clusters	2
Individual/organized	1
Individual/disorganized	0
Integration with surrounding cartilage	
Two edges	2
One edge	1
Without integration	0
Cartilage thickness	
2/3	2
1/3–2/3	1
<1/3	0
Subchondral bone	
Normal	3
Increasing remodelling	2
Bone necrosis/granulation tissue	1
Detached/fracture/callus at base	0

### Immunohistochemistry analysis

For IHC analyses, serial sections were stained for collagen type I and collagen type II (Santa Cruz Biotechnology, Inc., Santa Cruz, TX, USA). The paraffin-embedded sections were de-paraffinized and subsequently treated for 4 min. with proteinase 1 (Ventana Medical Systems, Inc., Tucson, AZ, USA). Sections were then stained using mouse anti-human collagen type I or goat anti-human collagen type II primary antibody diluted to 1:100 with TBST, followed by antimouse or anti-rabbit secondary antibody. The sections were exposed to diaminobenzine for ∽5 min. The sections were counterstained with haematoxylin for 30 sec. A positive reaction resulted in brown staining. The collagen type II staining areas were counted using the Image J program.

### Biomechanical analyses

**Figure 2:**
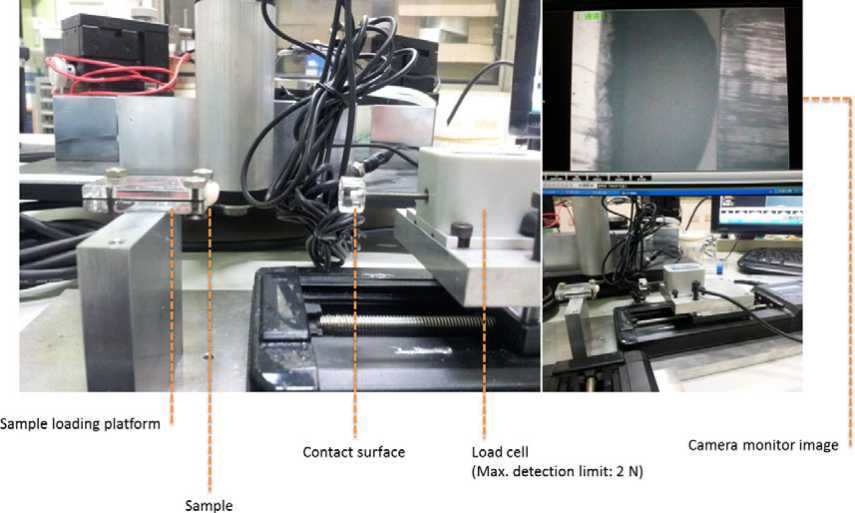
Video-assisted testing device for biomechanical compression testing of the condyles.

The specimens of the contralateral knee (*n* = 6) were immediately and under fresh conditions evaluated with regard to their biomechanical properties. In a specially prepared testing device (Fig.2), a compression testing of the positive control, the negative control as well as the flap-covered condyle was performed with regard to elasticity and maximum value (failure load).

The device we used was built by TiMMeL Lab from the Department of Mechanical Engineering of National Taiwan University. It was constructed of a sample loading platform (self-constructed, using stainless steel and polymethylmethacrylate parts), a stepping motor driven stage (product name: SGSP20-85(X); SIGMA KOKI Co., Ltd., Sumida-ku, Tokyo, Japan), a stage controller (product name: SHOT-204MS; SIGMA KOKI Co., Ltd.), a load cell (product name: LTS-200GA; Kyowa Electronic Instruments Co., Ltd., CHOFU-SHI, Tokyo, Japan) and data recorders/analysers (product name: DBU-120 A; Kyowa Electronic Instruments Co., Ltd.). Samples were fixed on the loading platform and compressed by the load cell located on the stepping motor driven stage. By the stage controller, the displacement of the load cell was controlled with a velocity of 10 μm/sec., which was directly related to the deformation of the samples. Force signals were measured by load cell and analysed by data recorders/analysers.

### Statistical analysis

The quantitative data were analysed and compared using SPSS 16.0 (IBM Corp., New York, NY, USA) statistical software by unpaired *t*-test. Statistical error α is sought to be 5% (alpha 0.05), so values with difference (*P* < 0.05) are to be considered significant.

## Results

### Macroscopical analyses

In gait analysis, four rabbits showed complete and natural walking pattern, two rabbits used one lower limb incompletely each showing a relieving posture.

All wounds healed completely without signs of infection. A slight swelling was noted in 10 of 12 legs.

The clinical testing showed all 12 joints to be stable in lateral and medial directions (collateral ligaments) and antero-dorsal directions (cruciate ligaments). The joints had a full range of motion in extension and flexion.

**Figure 3:**
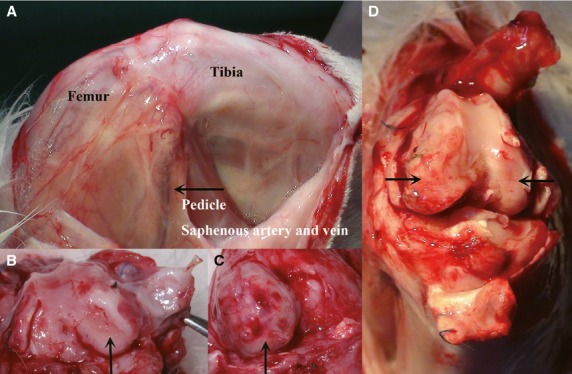
Macroscopic analysis after 4 weeks healing period. (A) View from the medial aspect of the knee, the arrow indicates the saphenous artery and vein pedicle, which showed strong pulsation (and therewith nourishment of the flaps) in all cases. (B and C) Views on the condyles after removal of the patella, showing both, a complete and a partial coverage of the defect with the periosteum flap. (D) Another case with complete integration of the periosteum flap into the defect, building a thin neo-cartilage layer with a smooth surface. The negative control showed to cartilage recovery signs, the donor site healed completely.

After reopening the surgical site, a complete macroscopic recovery of the donor site could be noted in all cases, the periosteal defects were covered by new tissue (which was not evaluated further; Fig.3A and D).

All 12 pedicles showed a strong and regular pulsation as first sign for perfusion and flap survival (Fig.3A). For specimen harvesting, the pedicles later had to be dissected and a strong pulsatile bleeding out of the saphenous vessels could be detected.

In five joints, we could detect a slightly laterally dislocated patellar tendon; in seven joints, the patellar tendon was still in its correct position.

After removal of the patella, flap attachment could be assessed. Five flaps were stable and completely attached to the defect surface, and seven flaps showed a partial attachment (6 × 50% coverage, 1 × 30% coverage; Fig.3B–D).

### Histological evaluation

**Figure 4:**
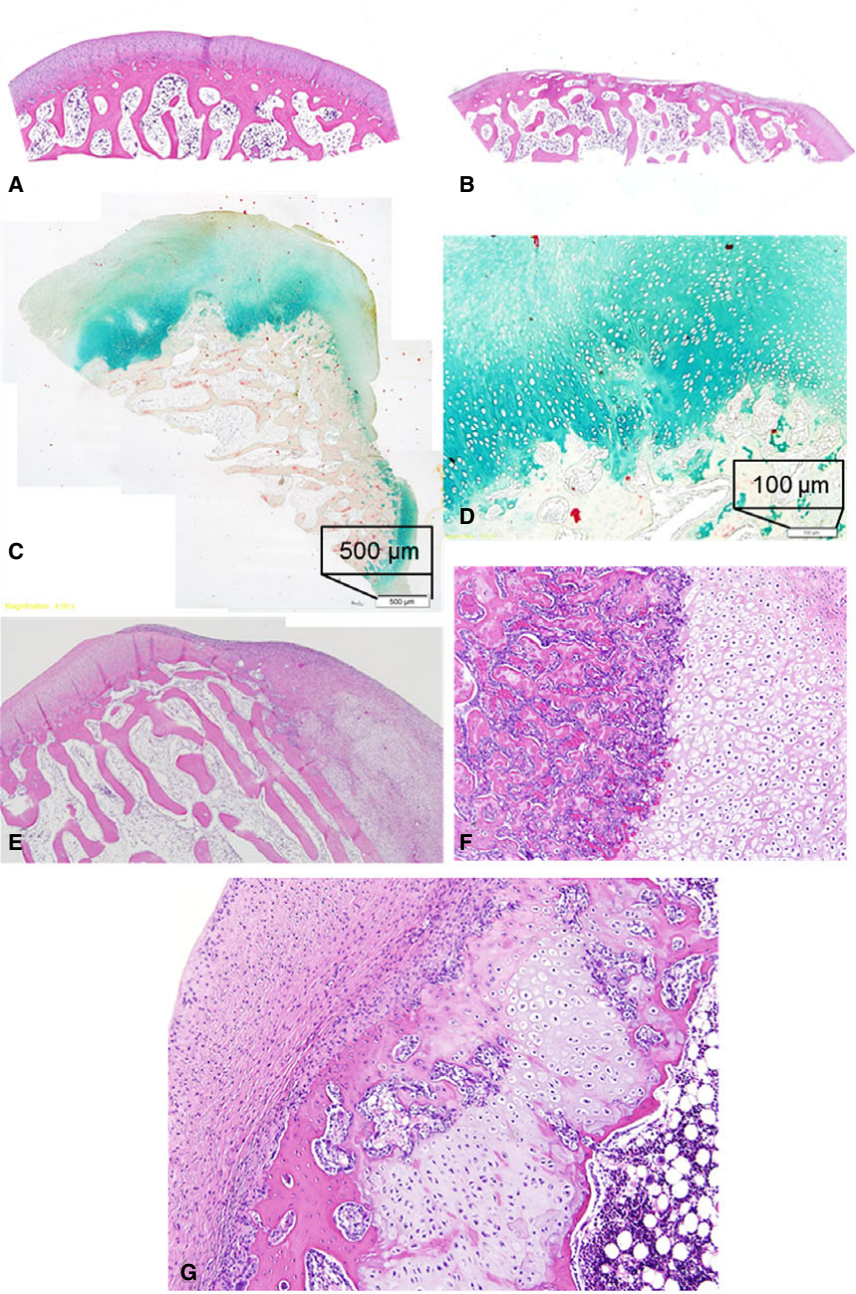
Histological evaluation. (A) Positive control, haematoxylin and eosin staining, 4×. (B) Negative control, HE, 4×. (C) Neo-cartilage built out of periosteum flap (Movat's staining). (D) Hyaline matrix and columnar cell distribution of the neo-cartilage in the attachment zone (Movat's staining). (E) Integration of the periosteum flap (right part), stable covering the bony surface and the surrounding original cartilage (left part), note the smooth surface of the flap. HE 4×. (F) Focus on stable attachment of flap, which already changed to neo-cartilage (right) to the bony surface (left). HE 10×. (G) Mixed composition of some periosteum flaps. The flap shows a strong attachment to the subchondral bone (right). It consists of newly formed cartilage at its base, close to the bone and still an overlying fibrous layer with a smooth regular surface. In between, a small island of new built bony tissue can be noted. HE 10×.

According to the ICRS scale (Table1) 11, the positive controls were characterized through a smooth and continuous surface, a hyaline matrix, columnar clusters and normal subchondral bone as are typical signs for healthy joint cartilage (Fig.4A). The negative controls still showed the cartilage defect without signs of cartilage recovery. The subchondral bony surface was just covered by thin layers of fibrous tissue (Fig.4B).

The histological evaluation of the six flap-covered medial femur condyles revealed the following: Concerning the cartilage thickness, it was in all cases higher than the surrounding original cartilage. In five knees, it was thicker than 2/3 of the normal cartilage and in one knee, it was up to 2/3 thicker than normal cartilage (Fig.4C). The tissue was composed of cartilage matrix at the basis, close to the bony surface, which was still covered by a fibrous matrix (Fig.4C and G). The surface was smooth in three cases and smooth with irregularities in three further cases. The integration of the flap tissue into the bony surface was in three cases complete (Fig.4E) and in three cases, only partially complete. In six cases, the matrix consisted of hyaline/fibrous mixed tissue, showing the cartilage-typical cell distribution of columnar clusters (Fig.4D, E and G). In three cases, small islands of newly formed bone tissue could be found in the histological sections (Fig.4G). The underlying subchondral bone showed some recovery signs and no signs of fracture, necrosis or callus formation.

### Immunohistochemistry

**Figure 5:**
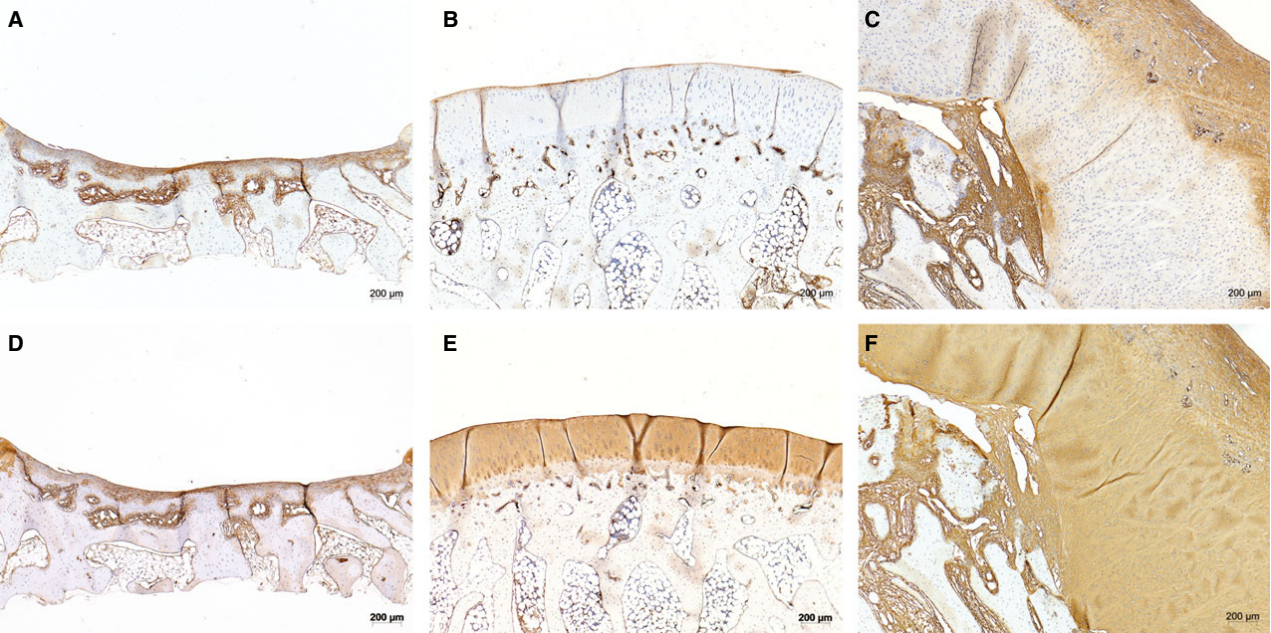
IHC staining with Coll I (upper row, A–C) and Coll II (lower row, D–F) of corresponding sections of negative control (A and D), positive control (B and E) and condyle covered with periosteum flap (C and F). Coll I staining is found mainly in fibrous tissue (*e.g*. of the periosteum flap) and bony surface (*e.g*. of the free subchondral bone in negative control), but not in cartilage, as typical. The original cartilage of the positive control as well as the neo-cartilage show characteristic positive results for Coll II.

Immunostaining for collagen type I and II showed specific staining patterns for the tissue subtypes of the sections (Fig.5, Table2). The normal articular cartilage of the positive control (and also of the borders of the negative control) as well as the neo-cartilage of the periosteum flap stained weakly for collagen type I, but strong for collagen type II. Collagen type I was more located in adjacent osseous tissue and stained intensely the fibrous tissue of the periosteum flaps and the thin layer on the negative control (Fig.5, Table2).

**Table 2:** Summary of immunochemical staining of the different tissue types in negative control group, positive control group and fibrous tissue with Collagen I and Collagen II

Tissue type	Coll I			Coll II		
	Bone	Cartilage	Fibrous tissue	Bone	Cartilage	Fibrous tissue
Negative control	+	−	+	−/+	+++	+
Positive control	+	−	nm	−	+++	nm
Periosteum flap	+	−	++	−/+	+++	++

The original cartilage as well as the neo-cartilage show characteristic positive results for Coll II, but not for Coll I, which is slightly expressed in bone and strong in fibrous tissue (nm: non-measurable, as there is no fibrous tissue located in the positive control sections). Please see also the pictures (Figure5).

### Biomechanical testing

**Figure 6:**
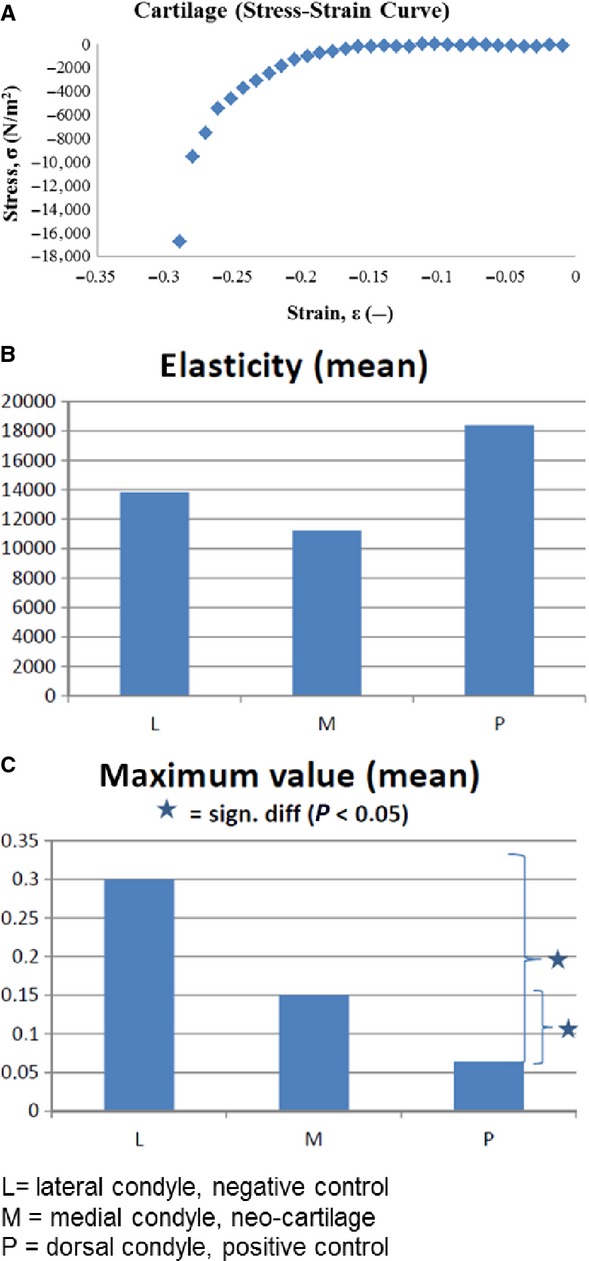
Biomechanical compression testing. (A) Exemplary Cartilage (Stress–Strain Curve) diagram, presenting the elasticity in the right linear part of the curve, the area of higher resistance in the more bended part and the maximum load at the sharp decline of the curve left. (B) Diagram of the elasticity (N/m^2^) of the three groups. (C) Diagram of the maximum load (MPa). (SPSS 16.0 statistical software, one-way anova and *post hoc*Tukey test (*P* < 0.05 is considered to be significant). For B and C: L = lateral condyle, negative control, M = medial condyle, neo-cartilage, P = dorsal condyle, positive control.

The results of the biomechanical compression testing are presented in Figure6. Starting point of the calculation were the ‘Cartilage (Stress–Strain Curve)’ diagrams, one of them is shown exemplarily in Figure6A. The left part of the curve shows an almost linear relationship, presenting the elasticity (calculated by the ascending gradient or slope of the straight line), where little stress results in higher material deformation. The left, more bent part of the curve represents the area of higher resistance, where increasing stress results in less deformation. The limit to the deformation can be seen in the sharp decline at the end of the curve (maximum value).

The mean elasticity of the neo-cartilage groups (M) is lower than that of the intact cartilage of the positive control group (P). Elasticity of the negative control group (L) was in between both groups. The maximum value, which means the end-point of deformation, is highest in the purely bony tissue of the negative control group and lowest in the normal cartilage of the positive control group, with the neo-cartilage group in between. The differences were significant between P and L, as well as between P and M (Fig.6B and C).

## Discussion

With this study, we intended to combine the knowledge of tissue engineering and the experience in the field of plastic surgery to address the traumatologic and orthopaedic disease of osteoarthritis.

There have already been several efforts using various models concerning this promising field of cartilage engineering by periosteal tissue, but there is one aspect missing in this recent scientific field and in medical literature. On the one hand, there are several *in vitro* models using cell culture or different bioreactors 6,12–14 for proliferation of periosteal chondrocytes. On the other hand, there are multiple *in vivo* models with different tissue transfer designs. All of them work with free non-vascularized periosteum transfers, always resulting in incomplete filling of the defect and presence of fibrous tissue instead of hyaline cartilage 9. As adequate nutrition of transferred tissue is an indispensable precondition, which in case of periosteal tissue may not only occur by synovial fluid, we considered a through blood supply nourished flap model to the necessary next step in joint cartilage repair research.

Thus, in this ‘translational’ approach, we evaluated for the first time in medical literature a vascularized periosteum flap as ‘independent’ biomaterial with its own blood supply to reconstruct circumscript cartilage defects of the knee.

As first and promising result of our study, we can assume to be on the right way with this hypothesis. As described in the results section, all flap pedicles showed a strong pulsation at the end-point of the experiment, as a sign of perfusion and stable nourishment of the transferred flaps. All flaps survived without any signs of necrosis or cell death; no avital tissue or infection was found. In addition, newly formed cartilage tissue was formed, filling up the created cartilage defect. Thus, the maintenance of an independent blood supply of the transferred periosteum *via* a dedicated pedicle may be a milestone in engineering of cartilage *in vivo*.

**Figure 7:**
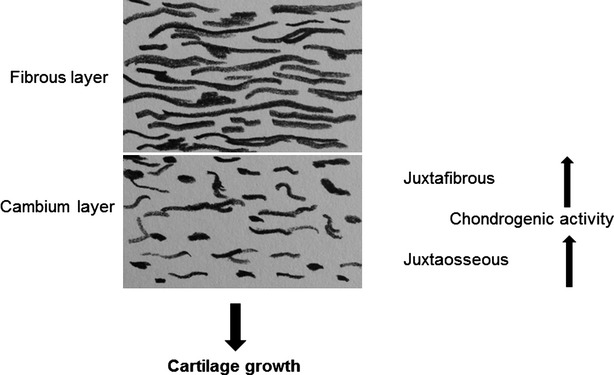
Composition of periosteum with its fibrous and cambium layer. Chondrogenic activity starts in the juxtaosseous area of the cambium layer and progresses to juxtafibrous area in the cambium layer. Growth of regenerated neo-cartilage is directional, away from the fibrous layer.

In the following, we want to go deeper into the details of the used model and our findings:

Periosteum consists of two discrete layers: the inner cambium layer containing the undifferentiated MSCs 15 and chondrocyte precursor cells and the outer fibrous layer (Fig.7). The cambium layer is connected to the bony surface *via* a juxtaosseous area and to the fibrous layer *via* a juxtafibrous area 1. *In vitro* and *in vivo* experiments have proven cartilage formation originating from the periosteum and found out that this cartilage formation and chondrogenesis commence in the juxtaosseous area of the cambium layer and that neo-cartilage growth is appositional, away from the fibrous layer 1. During chondrogenesis, the fibrous layer persists while neo-cartilage progresses from the juxtaosseous region to the juxtafibrous region of the cambium layer and gradually replaces it. In our results, we can underline these findings. New cartilage was built within the observation time of 4 weeks and was found to be located at the base of the flap in the attachment zone to the bone which is represented by the juxtaosseous area of the cambium layer. The fibrous layer persisted and covered the neo-cartilage with a smooth surface (Fig.4). It could be characterized by the strong expression of collagen type I in contrast to the cartilage tissue, which showed no collagen type I staining (Fig.5, Table2).

Regarding the time course of events in periosteal chondrogenesis, Miura *et al*. have determined a general sequence and time course of events that characterize chondrogenesis based on studies involving 850 periosteal explants from 2-month-old rabbits cultured for 6 weeks 16. Proliferation (3H-thymidine incorporation), which is the first event, was maximal on day 3 and decreased to baseline by day 10. Matrix synthesis was indicated by aggrecan expression that begins to rise on days 3–7, type II collagen expression that rises on days 7–10, and 35S-sulphate incorporation that began to rise on day 10. On day 21, a peak was reached for expression of aggrecan (sixfold), type II collagen expression (200-fold) and 3H-thymidine uptake. Type II collagen content increased after 14 days to plateau by 42 days. Histological evidence of chondrogenesis started by day 14 and reached a plateau by days 28–42. The explants stimulated by TGF-β1 increased in wet weight for 3 months. The current authors hypothesize that the development and maturation of neochondrocytes occurs in these three sequential phases that are distinguished by transition-restriction points and regulated separately by growth factors (and possibly mechanical or other factors). On the basis of these results, we assessed the observation time in our study to be 28 days to be sure to find histological evidence of neo-cartilage tissue. Our findings correspond precisely with the time course described, histologically visible cartilage formation was found after 28 days, although we of course only assessed this single point in time at week 4 in our whole healing period. In further studies, we plan to examine the cartilage growth in long-term period of 8 weeks to evaluate the question, whether the whole periosteum flap, including the fibrous layer will be completely replaced by newly formed cartilage.

It has to be considered, that biomechanical factors may affect periosteal chondrogenesis. Numerous studies showed that articular cartilage repair in rabbits, with or without periosteal grafts, improve with the use of post-operative continuous passive motion 2,17–19. Possible explanations could relate to oscillations in intra-articular hydrostatic pressure (dynamic fluid pressure), direct mechanical compression, fluid flow, and nutrient effects or shear forces. Continuous passive motion facilitates rapid clearance of a haemarthrosis because of cyclical oscillations in intra-articular pressure. Also for differentiation of the periosteal MSCs and precursor cells into chondrocytes, we considered such highly specific conditions, being typical for the ‘target tissue’ (like biomechanical activities for joint cartilage) to be essential. That is why we allowed the animals to move and weight-bear their joints freely.

The results also showed that with the developed surgical technique, a sufficient fixation of the flap onto the defect is possible. Although there were cases with partial attachment of the flap onto the bone observed, which may be because of incomplete integration on cellular level and a too short observation time, there was no flap dislocation through joint movements found. Of course, as also was shown in studies by O'Driscoll *et al*. and Rubak *et al*. 2,20, the depth of the defect into which a periosteal graft is placed is important. The defect must be deep enough to prevent compression of the graft surface through the opposite joint surface before extracellular matrix is deposited; and permit growth of the neo-cartilage so that it is not subjected unreasonable forces. Another aspect supporting the feasibility of the surgical technique is the fact that all joints were stable (collateral ligaments and cruciate ligaments) and had a full range of motion. Except for two rabbits, who presented a slight ‘hobbling’ with one leg, the animals used their legs normally and without pain restriction. The defects of the negative controls in addition showed no signs of cartilage recovery, they were only covered by a thin layer of collagen type I positive fibrous tissue. This proofs that their size was large enough (critical size) and the cartilage formation was not supported from the subchondral tissue.

Focussing on our histological results, several aspects need to be discussed. First, as already mentioned, newly formed cartilage tissue was found as expected in the juxtaosseous area of the cambium layer. According to the Scoring of the ICRS 11, this was characteristic and healthy cartilage tissue consisting of hyaline matrix and columnar cell distribution. The IHC staining results underline these findings with a characteristic distribution of weak staining with collagen type I, but strong staining with collagen type II of the cartilage. At the assessed time-point of 4 weeks healing, the periosteum flap was not completely replaced by new cartilage. The cartilage was still covered by a fibrous layer. In addition, the whole composite was up to 2/3 thicker than the surrounding normal cartilage. In further long-term experiments, we plan to observe the further changes.

Another important finding is that the periosteum is able to strongly attach to the subchondral bone, however in some cases it attached only partially. On the one hand, this again supports the feasibility of the surgical technique of transosseous sutures and tissue glue fixation. On the other hand it shows that a complete integration of the transplant on cellular level is possible despite full weight bearing and free movement.

Concerning the surgical fixation technique, we consider the role of the tissue glue to be special. Adhesives like the used fibrin sealant TissueCol® are known to offer more benefits and enhancements to tissue healing than fixation, including improved biocompatibility, resorbability and non-immunogenicity 21. As a bioactive agent, it is able to support cartilage regeneration in terms of chondrocyte survival 22 and their secretion of extracellular matrix 23 and is described to be a promising biomaterial for articular cartilage tissue engineering itself. In our study, we used the fibrin glue for both purposes, fixation and bioactive agent, but the exact contribution of the glue to our results has to be addressed in further studies (see limitations of the study).

Moreover, in some specimens, small islands of bone tissue were found between the neo-cartilage and the fibrous layer of the periosteum flap. This may be because of a differentiation of some MSCs into osteoblasts. Of course, periosteum is also a widely used and well-known model for bone growth support and bone reconstruction 24,10 and differentiation into osteogenic cells. While designing our study, we assumed that the periosteal stem cells, always differentiating into the direction of the specific condition surrounding them, will detect the joint-typical factors like synovial fluid and growth factors, compression and sliding load to differentiate into cartilage. The fact that mainly cartilage was formed supports our assumption. For the next studies, we aim to look at this bone formation in more detail (especially focusing on long-term observation).

The biomechanical results reflect our histological findings. The elasticity was highest in the normal cartilage of the control group, but lowest in the neo-cartilage group, which was even lower than the negative control group. This may be explained by the very heterogenous tissue of the periosteum flap. Only one-third of the flap at a maximum consisted of cartilage tissue. This may be because of the simultaneously present bony islands and fibrous tissue which have different biomechanical properties. The maximum value shows a more clear and comprehensive result. It was highest in the purely bony tissue of the negative control and significantly low in the normal cartilage of the positive control group. The mixed composition of the periosteum flap group led to values in between both, being significantly higher than the positive control.

Of course our study has its limitations, being a pilot study and only working with six rabbits in total. Although we designed a positive control and a negative control, a comparison with a group of a free, non-vascularized flap would complete the design. Also the role of the used tissue glue for flap fixation and attachment has to be addressed further; a control group not using the tissue glue would be useful. In addition, more animals would be necessary to achieve statistically valid results. As already mentioned, our next step will be a long-term study assessing the changes in the periosteum flap after a minimum 8 weeks’ time period.

In the present study, we wanted to examine the surgical technique of a vascularized periosteum flap and its feasibility for the first time. For this pilot experiment, we used young, 3-month-old animals, which of course do not represent the elderly patient, who might have a local osteoarthritis. Only circumscript traumatic cartilage defects may be represented through this model. It is known that the chondrocyte precursor cells vary in total density and volume with age and in different donor sites 8, so the use of older animals would be another important step.

In conclusion, this study showed that a vascularized periosteum flap is able to generate cartilage. A reconstruction of circumscript cartilage defects seems to be possible. Although these are first results out of a pilot study, this technique, we believe, can have a wide range of potential applications such as the development of naturally derived biomaterials, cartilage tissue engineering, as well as regenerative medicine.
